# The JAK1/2 inhibitor ruxolitinib delays premature aging phenotypes

**DOI:** 10.1111/acel.13122

**Published:** 2020-03-20

**Authors:** Audrey Griveau, Clotilde Wiel, Dorian V. Ziegler, Martin O. Bergo, David Bernard

**Affiliations:** ^1^ Centre de Recherche en Cancérologie de Lyon Inserm U1052 CNRS UMR 5286 Centre Léon Bérard Université de Lyon Lyon France; ^2^ Department of Biosciences and Nutrition Karolinska Institutet Huddinge Sweden

**Keywords:** cellular senescence, JAK/STAT pathway, progeria, ruxolitinib

## Abstract

Hutchinson–Gilford progeria syndrome (HGPS) is caused by an *LMNA* mutation that results in the production of the abnormal progerin protein. Children with HGPS display phenotypes of premature aging and have an average lifespan of 13 years. We found earlier that the targeting of the transmembrane protein PLA2R1 overcomes senescence and improves phenotypes in a mouse model of progeria. PLA2R1 is regulating the JAK/STAT signaling, but we do not yet know whether targeting this pathway directly would influence cellular and in vivo progeria phenotypes. Here, we show that JAK1/2 inhibition with ruxolitinib rescues progerin‐induced cell cycle arrest, cellular senescence, and misshapen nuclei in human normal fibroblasts expressing progerin. Moreover, ruxolitinib administration reduces several premature aging phenotypes: bone fractures, bone mineral content, grip strength, and a trend to increase survival in a mouse model of progeria. Thus, we propose that ruxolitinib, an FDA‐approved drug, should be further evaluated as a drug candidate in HGPS therapy.

## Graphical abstract

In this study, we have described that inhibition of JAK1/2 by ruxolitinib improves some progeria phenotypes in normal human fibroblasts and in a murine model of progeria. Our findings should encourage the design of new studies to explore the potential clinical benefits of treating by ruxolitinib, alone or in combination, HGPS patients.

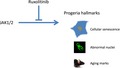

Hutchinson–Gilford progeria syndrome (HGPS) is a lethal premature aging disorder. HGPS is caused by a mutation in the *LMNA* gene, leading to the production of progerin, a truncated prelamin A form, which induces misshapen nuclei, cellular senescence, and aging (Goldman et al., 2004). The functional involvement of cellular senescence in promoting aging and age‐related pathologies has been confirmed during the last few years (Childs et al., 2017).

The Janus kinase (JAK)/signal transducer and activator of transcription (STAT) pathway regulates cellular senescence and some age‐associated alterations, as evidenced by the use of the JAK1/2 inhibitor ruxolitinib, an FDA‐approved drug used in the management of myeloproliferative disorders, which decreased bone loss and adipose tissue inflammation and improved physical condition during aging in mice (Farr et al., 2017; Xu et al., 2015; Xu, Tchkonia, & Kirkland, 2016). These effects of ruxolitinib have been attributed to the inhibition of the senescence‐associated secretory phenotype (SASP) (Farr et al., 2017; Xu et al., 2015, 2016).

The phospholipase A2 receptor (PLA2R1) is a transmembrane protein able to interact with some secreted PLA2 (sPLA2), collagens, and integrins. However, little is known about the role of this protein and of these interactions. PLA2R1 promotes cellular senescence in different contexts, and JAK/STAT signaling mediates the pro‐senescent effects of PLA2R1 (Augert et al., 2009; Bernard & Vindrieux, 2014; Griveau et al., 2018; Vindrieux et al., 2013). Consistently, the JAK/STAT pathway drives the cell cycle arrest associated with PLA2R1‐induced senescence, thus affecting senescence beyond the sole inhibition of the SASP as described by others (Farr et al., 2017; Xu et al., 2015, 2016) or/and because the SASP can be also a critical mediator of the full senescence (Acosta et al., 2008). Moreover, we recently observed that PLA2R1 contributes to progeria phenotypes in vitro in progerin‐expressing cells and in vivo in the *Zmpste24*−/− murine model of progeria (Griveau et al., 2018).

These results raise the interesting possibility that targeting JAK/STAT signaling directly might overcome senescence and reduce disease phenotypes in progeria. In this study, we defined the impact of ruxolitinib, an FDA‐approved JAK1/2‐competitive inhibitor that binds to and inhibit their kinase activities (Mascarenhas & Hoffman, 2012), on the development of senescence and premature aging phenotypes induced by mutant prelamin A.

To define the ability of ruxolitinib to influence progerin‐induced senescence, we used normal human fibroblasts (MRC5) constitutively expressing progerin or control cells expressing lamin A (Griveau et al., 2018). The expression of progerin strongly inhibited cell proliferation when compared to lamin A‐expressing cells (Figure 1a,b). The addition of ruxolitinib prevented the progerin‐associated growth inhibitory effect (Figure 1a,b). As expected, the constitutive expression of progerin induced premature senescence, as evidenced by an increase in senescence markers, such as senescence‐associated β ‐galactosidase staining (SA‐β‐Gal), increased expression of the cyclin‐dependent kinase inhibitors (CKI) CDKN1A and CDKN2A, and increased expression of the IL‐8 SASP component (Figure 1c‐f). Strikingly, all of these senescent characteristics were abolished upon ruxolitinib treatment (Figure 1c‐f). Moreover, similar results were obtained using HGPS‐derived fibroblasts in which ruxolitinib increased the replicative potential and decreased several senescence markers (Figure S1). Hence, the JAK1/2 inhibitor ruxolitinib inhibits progerin‐induced senescence in normal human fibroblasts.

**Figure 1:**
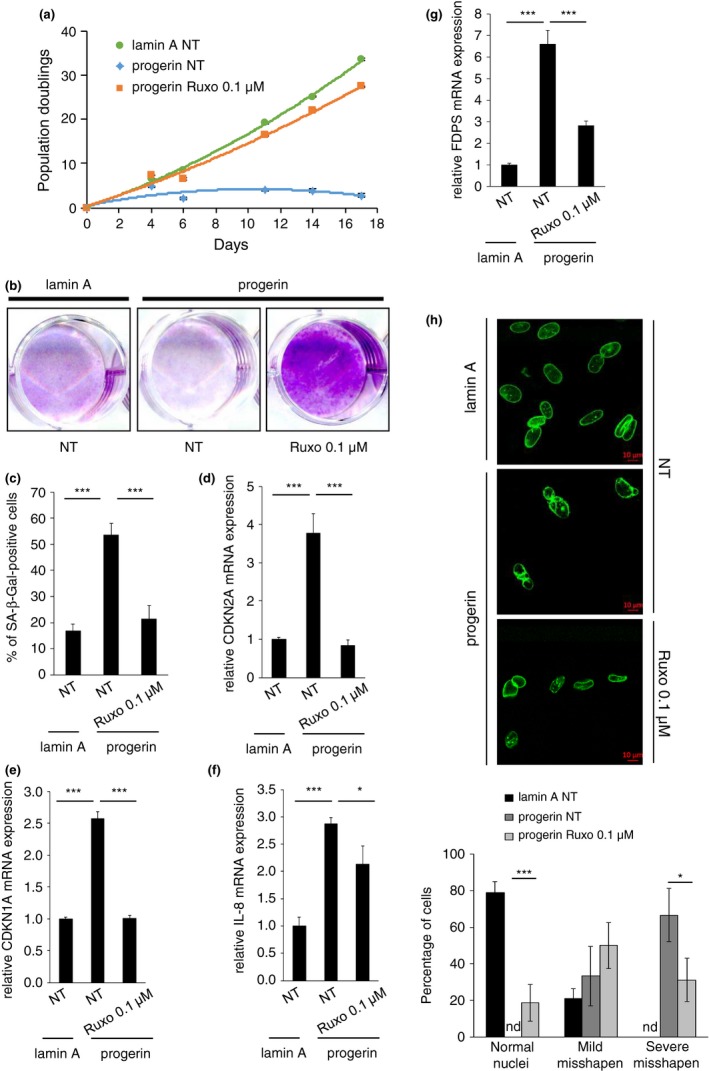
Ruxolitinib prevents progerin‐induced senescence. MRC5 cells were infected with retroviral vectors encoding lamin A or progerin, and subsequently, puromycin was selected. Cells were then treated every 2 days with ruxolitinib at 0.1 µM. (a) Growth curves were performed by counting and seeding the same number of cells at each passage. Cell population doubling was calculated. (b‐c) Twelve days after the beginning of the treatment, the same number of cells was seeded, (b) fixed and stained by crystal violet, or (c) assessed for SA‐β‐Gal activity. (d‐g) RNAs were isolated 15 days after the beginning of the treatment and reverse‐transcribed, and RT–qPCR was performed for CDKN2A, CDKN1A, IL‐8, or FDPS. Results were normalized against ACTB. (h) Fifteen days after the beginning of the treatment, cells were analyzed by confocal microscopy. Representative pictures are shown, and counting of normal, mildly misshapen, and severely misshapen nuclei was performed. Nd means not detected. Results presented in this figure are representative of at least two independent experiments. Error bars indicate *SD* of measurements taken in triplicate. Statistical analysis was performed with Student's *t* test (*p < .05; **p < .01; ***p < .005)

Earlier studies revealed that PLA2R1 contributes to progerin‐induced senescence by stimulating farnesyl diphosphate synthase (FDPS) expression and misshapen nuclei formation (Augert et al., 2009; Griveau et al., 2018; Vindrieux et al., 2013). FDPS controls the production of a farnesyl lipid, which is attached to the carboxyl terminus of progerin and which is required for its ability to cause disease and induce the characteristic nuclear shape abnormalities observed in HGPS cells (Varela et al., 2008). We argued that if JAK/STAT signaling mediates PLA2R1‐induced senescence in progerin‐expressing cells, ruxolitinib should prevent those phenotypes. Indeed, that was the case: Ruxolitinib reduced the high level of FDPS expression and misshapen nuclei in progerin‐expressing cells (Figure 1g,h), without changing GFP‐progerin protein level (Figure 1h and Figure S2). Overall, these results confirm that JAK1/2 inhibition by ruxolitinib impacts not only the secretome of senescent cells, as already suggested by others (Farr et al., 2017; Xu et al., 2015, 2016), but also the cell cycle arrest, which can itself be induced by the SASP (Acosta et al., 2008).

Next, we determined whether ruxolitinib treatment could affect progeria‐like phenotypes in vivo. Full‐length prelamin A accumulates in cells of *Zmpste24*‐deficient mice, which triggers nuclear shape abnormalities and several hallmarks of progeria, including bone abnormalities, rib fractures, muscle weakness, low body weight, and premature death (Bergo et al., 2002). Ruxolitinib administration reduced the number of rib fractures and increased grip strength, bone mineral content (BMC), and survival (Figure 2a‐d). These results represent the first proof of concept in cells and mice for the use of ruxolitinib in the treatment of premature aging syndromes.

**Figure 2:**
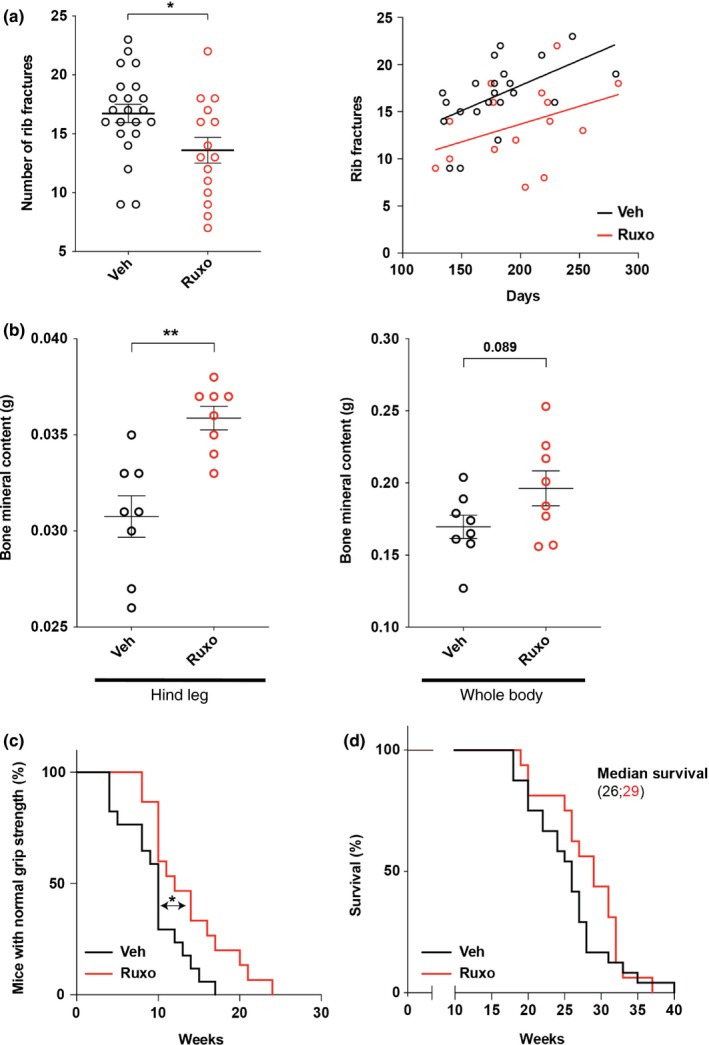
Ruxolitinib delays premature aging phenotypes. (a) Left, number of rib fractures in *Zmpste24*−/− mice treated with vehicle (*n* = 22) or ruxolitinib (*n* = 15) at time of death. Right, plot showing the number of rib fractures (y‐axis) and survival (x‐axis) and the linear regressions obtained for each condition. (b) Dual‐energy X‐ray absorptiometry (DEXA) analyses of the bone mineral content (BMC) from mice at time of death are shown. (*n* = 8, five females and three males; per group, average age of mice analyzed is 26.75 weeks and 27.875 weeks for vehicle‐ or ruxolitinib‐treated groups, respectively). (c) Kaplan–Meier plot showing the percentage of *Zmpste*24−/− vehicle‐treated (*n* = 24) and *Zmpste24*−/− ruxolitinib‐treated (*n* = 16) mice with normal grip strength. (d) Kaplan–Meier curves showing survival of vehicle‐treated and ruxolitinib‐treated mice (*n* = 24 and *n* = 16, respectively). Errors bars indicate *SEM*. Statistical analyses were performed using the two‐sided Student's *t* test for (a) and (b) and the log‐rank test

The effect of ruxolitinib on progeria phenotypes is a phenocopy of that observed in *Pla2r1* knockout mice, that is, reduced numbers of rib fractures, increased BMC, and improved grip strength (Griveau et al., 2018). Moreover, similar to *Pla2r1*−/− mice, ruxolitinib did not affect body weight or bone mineral density (BMD) (Figure S3a,b). These results support the idea that JAK1/2 mediates PLA2R1‐induced cellular senescence corroborating our previous findings (Griveau et al., 2016; Vindrieux et al., 2013). Even if unusual, increased BMC without increased BMD has already been reported in human and mouse studies (Andreassen & Oxlund, 2001; Khamoui et al., 2017; Van Loan, Johnson, & Barbieri, 1998; Windahl, Vidal, Andersson, Gustafsson, & Ohlsson, 1999), and this can eventually occur in the context of weight loss (Khamoui et al., 2017; Van Loan et al., 1998), the murine model of progeria used in our study losing weight. This increase in BMC without increase in BMD was explained by increased bone area (Figure S3c).

We administered ruxolitinib in subcutaneously implanted slow‐release pellets. Advantages of this system are that it delivers drug continuously over a long period of time and it reduces the daily stress associated with traditional drug delivery approaches. A potential disadvantage is that drug doses will likely be lower than with traditional methods. Hence, it is possible that the effects of ruxolitinib observed in the current study are underestimated. This argument could conceivably explain the robust effects of ruxolitinib on cultured cells and the relatively modest effects in vivo. Regardless, some improvements in disease phenotypes in vivo were statistically significant and reproducible. Overall, we believe our study should encourage the design of comprehensive preclinical trials to adjust dosage and routes of administration in progerin‐knock‐in mice, such as the Lmna^G609G/G609G^ mouse model, which more faithfully recapitulates phenotypes of HGPS, alone or in combination with compounds such as farnesyl transferase inhibitor (FTI) (Gordon et al., 2016, 2012). Indeed, our results support that ruxolitinib, by inhibiting FDPS expression that is upstream of FT, impacts the same pathway than FTI. They may then cooperate to revert progerin effects.

Ruxolitinib is currently used as an antitumor agent in the context of myeloproliferative disorders and is proposed to decreased inflammation (Bose & Verstovsek, 2017; Ghoreschi & Gadina, 2014; Lussana & Rambaldi, 2017), in agreement with previous observations that ruxolitinib represses pro‐inflammatory SASP production (Farr et al., 2017; Xu et al., 2015, 2016). Although the use of ruxolitinib is relatively safe, there are reports of reduced blood cell counts and increased risk of infection and nonmelanoma skin cancer, which would be important to consider before testing in HGPS patients (Fabiano et al., 2015; Saeed, McLornan, & Harrison, 2017). Beyond ruxolitinib and JAK inhibition, another key mediator of the pro‐inflammatory SASP, namely the NF‐kB transcription factor, is also an important mediator of HGPS (Osorio et al., 2012). Taking into account the lack of current therapeutic opportunities for the devastating HGPS disease, ruxolitinib, possibly in combination with other validated drugs, could thus constitute a new strategy (Gordon et al., 2016, 2012) to improve the health of children with HGPS.

## CONFLICT OF INTEREST

None declared.

## AUTHOR CONTRIBUTIONS

AG, CW, and DVZ performed experiments. AG, CW, and DVZ analyzed experiments. MOB and DB designed the study. CW and DB wrote the manuscript with the contribution of all coauthors. MOB and DB supervised the study.
