# Supplementary material for: The JAK1/2 inhibitor ruxolitinib delays premature aging phenotypes
Source: Aging Cell. 2020 Mar 20;19(4):e13122. doi: 10.1111/acel.13122 (PMC7189991; doi:10.1111/acel.13122)
Supplement: Supplementary file 1 [file ACEL-19-e13122-s001.pdf]

Supplemental Figure 1

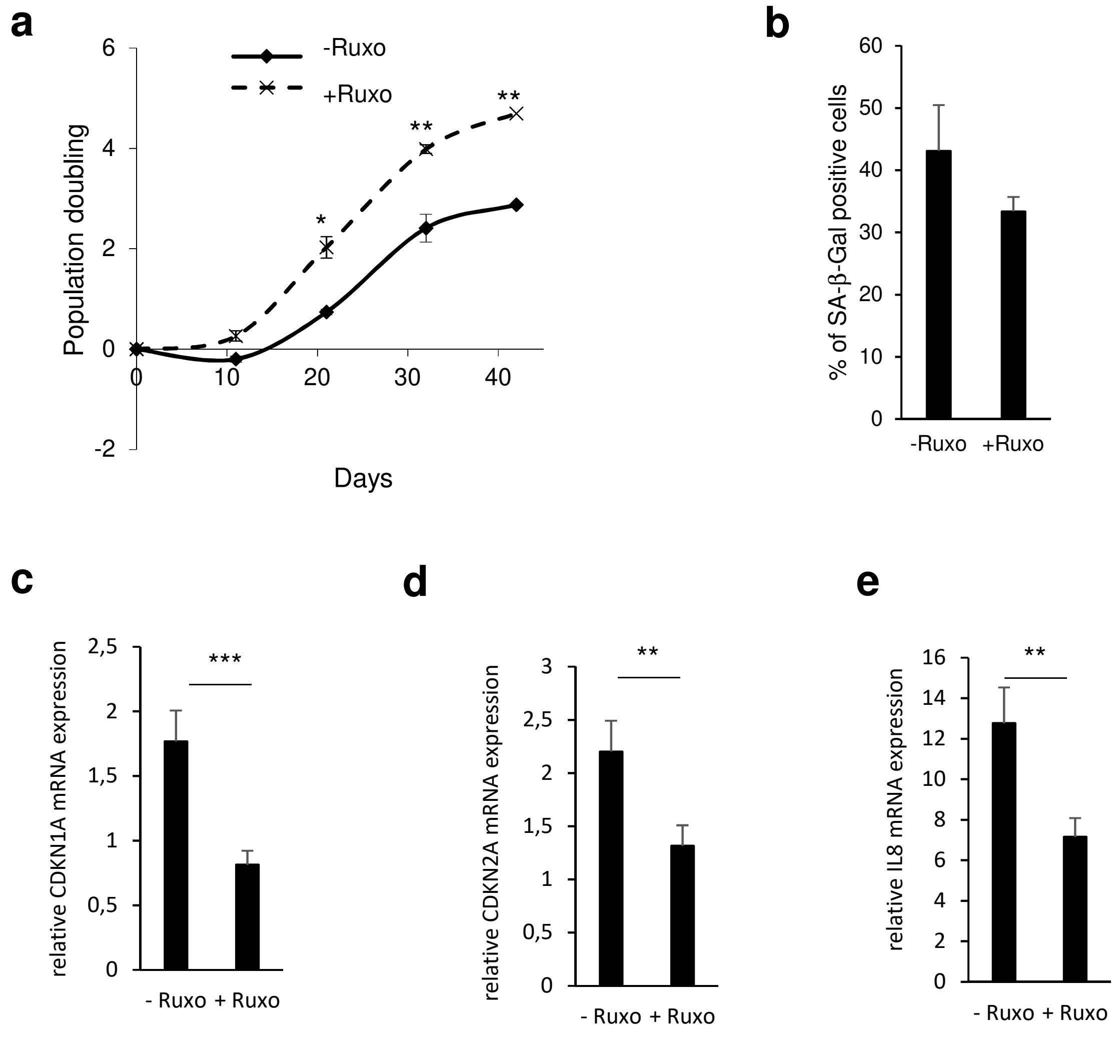

Figure S1 Ruxolitinib delays replicative senescence in HGPS-derived fibroblasts. (a) Cells were treated or not every 2 days with 0.1μM of Ruxolitinib. Growth curves were performed and cumulative population doubling displayed. (b) SA-b-Gal staining were performed after 30 days of treatment. (c-e) RNAs were prepared 30 days after the beginning of the treatment, reverse transcribed, and RT-qPCR were performed for CDKN1A, CDKN2A and IL8. Results were normalized using GAPDH. Error bars indicate SD of measurements taken in triplicate. Statistical analysis was performed with the Student's t test (* $P < 0.05$; ** $P < 0.01$; *** $P < 0.005$).

Supplemental Figure 2

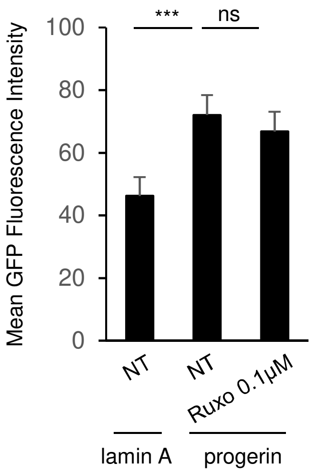

Figure S2 Ruxolitinib does not affect levels of progerin. MRC5 cells were infected with retroviral vectors encoding lamin A-GFP or progerin-GFP, selected and then treated every 2 days with Ruxolitinib at 0.1 µM. Fifteen days after the beginning of the treatment, cells were analyzed by confocal microscopy and GFP fluorescence intensity of nuclei quantified with ImageJ Software. Data are presented as mean fluorescence of 10-15 fields +/- SEM. Statistical analysis was performed with the Student's t test (*** P < 0.005).

Supplemental Figure 3

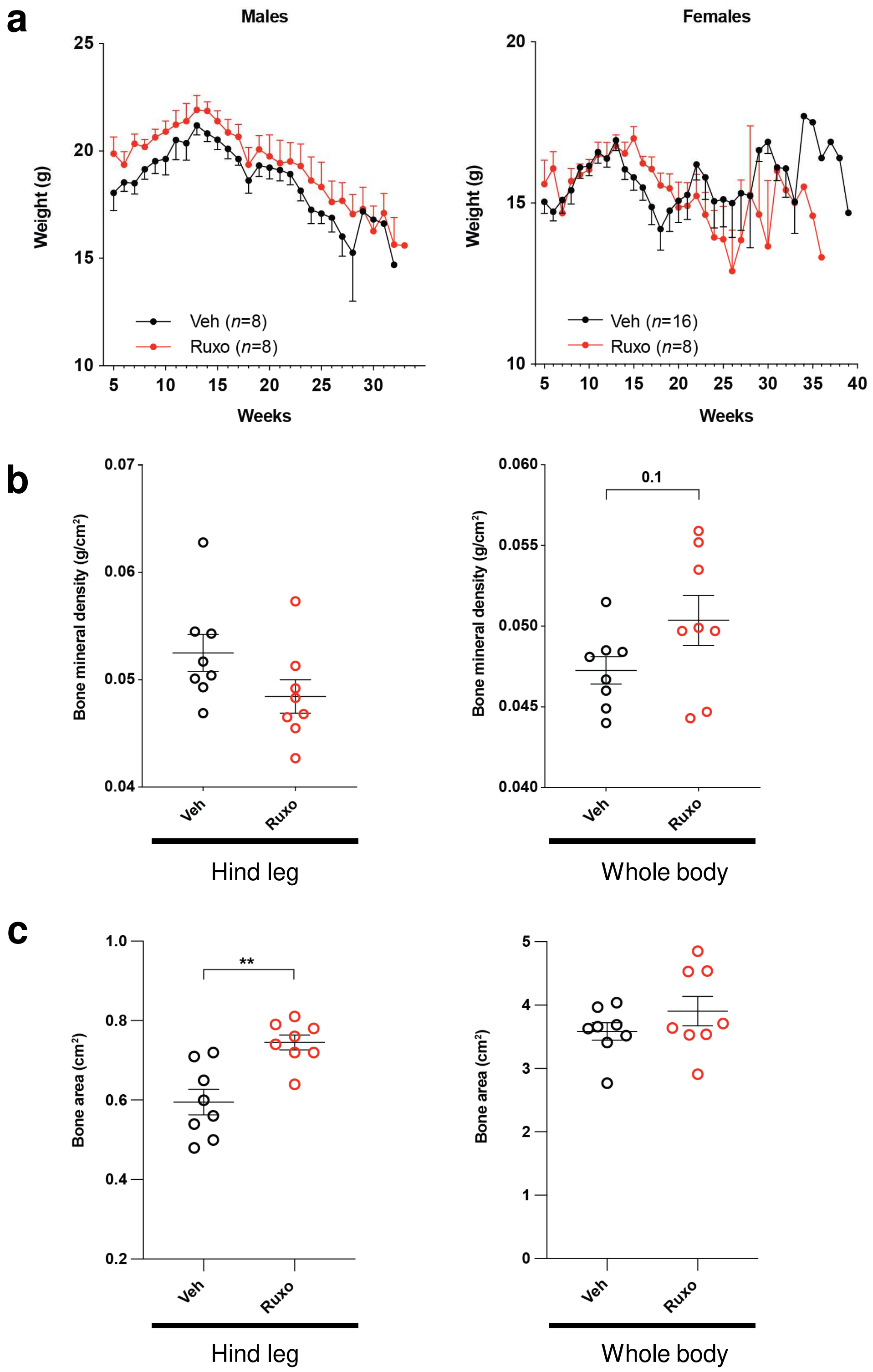

Figure S3 Ruxolitinib does not impact weight and bone mineral density of Zmpste24-/- mice. (a) Weight curves of Zmpste24-/- treated with vehicle or with Ruxolitinib. Left, males; right, females. (b-c) Dual-energy X-ray absorptiometry (DEXA) analyses of the bone mineral density (BMD) and bone area from mice at time of death are shown. (n = 8; 5 females and 3 males; per group, average age of mice analyzed is 26.75 weeks and 27.875 for vehicle or Ruxolitinib treated groups, respectively). Left, data obtained from hind leg; Right, data from whole-body.
