# Supplementary material for: The JAK1/2 inhibitor ruxolitinib delays premature aging phenotypes
Source: Aging Cell. 2020 Mar 20;19(4):e13122. doi: 10.1111/acel.13122 (PMC7189991; doi:10.1111/acel.13122)
Supplement: Supplementary file 2 [file ACEL-19-e13122-s002.pdf]

Online Supporting Information

Experimental procedures

Cell culture and reagents

MRC5 normal human fibroblasts (ATCC, Manassas, VA, USA), dermal fibroblasts from patient with HGPS carrying the 1824 C > T mutation were obtained from the NIA Aging Cell Culture Repository (AG03199; New Jersey, USA) and virus producing GP293 cells (Clontech, Mountain View, CA, USA) were cultured in Dulbecco′s modified Eagle′s medium (DMEM, Life Technologies, Carlsbad, USA) containing GlutaMax and supplemented with 10% FBS (Sigma-Aldrich, Saint-Louis, USA) and 1% penicillin/streptomycin (Life Technologies). Cells were maintained at 37°C under a 5% CO_2 atmosphere. Ruxolitinib (HY-50856, Clinisciences, Nanterre, France) was used at 0.1 µM.

Vectors, transfection and infection

The following vectors were supplied by Addgene: pBABE-puro-GFP-wt-lamin A (#17662), pBABE-puro-GFP-progerin (#17663). Virus-producing GP293 cells were transfected with vectors using the GeneJuice reagent according to the manufacturer's recommendations (Merck Millipore). Five million cells seeded in 10-cm dishes were transfected with the VSVg (1 µg) and the retroviral vector of interest (5 µg). Two days post-transfection, the viral supernatants were mixed with fresh medium (1/2) and hexadimethrine bromide (8 µg/ml; Sigma-Aldrich), and was then used to infect MRC5 cells for 6 h. One day post-infection, puromycin selection was initiated (500 ng/ml).

Senescence-associated β-Galactosidase (SA-β-Gal), crystal violet staining, and growth curves

For senescence-associated-β-Galactosidase assays, cells were washed twice with PBS, fixed for 5 min in 2% formaldehyde/0.2% glutaraldehyde, rinsed twice in PBS, and incubated at 37°C overnight in SA-β-Gal solutions as described (Augert et al. 2009). For crystal violet staining, cells were seeded onto 6-well plates, prior to being stained 12 days later as previously described (Augert et al. 2009). For growth curves, selected cells were seeded at the same density, split as indicated and counted. The population doubling was calculated at each passage.

RNA extraction, reverse transcription, and real-time quantitative PCR

RNA was extracted with phenol-chloroform using Upzol (Dutscher, Brumath, France). The Maxima First cDNA Synthesis Kit (Life Technologies) was used to synthesize cDNA from 1 µg of total RNA. The reverse transcription (RT) reaction mixture was diluted 1/20 and used as cDNA template for quantitative PCR (qPCR) analysis. TaqMan qPCR analyses were carried out on a FX96 Thermocycler (Biorad, Hercules, USA). The PCR mixture contained TaqMan mix (Roche, Boulogne-Billancourt, France), 200 nM of primers, the Universal Probe Library probe (100 µM) for the gene of interest (TaqMan Gene Expression Assays (Primers/probe); Life technologies) and 1.67 µl cDNA template. Reactions were performed in triplicate. The relative amount of mRNA was calculated using the Comparative Ct (ΔΔCT) method, following data normalization against actin for housekeeping genes. Sequence of the primers used were described previously (Griveau et al. 2018). Sequences of the primer used are: CDKN1A, fwd 5'-TCACTGTCTTGTACCCTTGTGC-3', rev 5'-GGCGTTTGGAGTGGTAGAAAT-3', UPL probe 32; CDKN2A, fwd 5'-GTGGACCTGGCTGAGGAG-3', rev 5'- CTTTCAATCGGGGATGTCTG-3', UPL probe

34; IL8, fwd 5'-AGACAGCAGAGCACACAAGC-3', rev 5'-ATGGTTCCTTCCGGTGGT-3', UPL probe 72; FDPS, fwd 5'-GAGTACCCGCCAACAAGC-3', rev 5'-ATCTAACCAGCGGGACA-3', UPL probe 15; GAPDH, fwd 5'-AGCCACATCGCTCAGACAC-3', rev 5'- GCCCAATACGACCAAATCC-3', UPL probe 60; ACTB, fwd 5'- ATTGGCAATGAGCGGTTC-3', rev 5'-GGATGCCACAGGACTCCAT-3', UPL probe 11.

Quantification of misshapen nuclei

MRC5 expressing lamin A-GFP or progerin-GFP vectors treated or not with Ruxolitinib were seeded in 8-well chamber slide plates (Dutscher). The cells were analyzed with a Zeiss LSM 780 NLO confocal microscope using a 63× oil-immersion objective. Images were captured using the Zen software. Nuclei were classified according to their shape: normal, mildly misshapen or severely misshapen. At least 200 nuclei were counted for each condition.

Mice and Ruxolitinib treatment

Mice knockout for *Zmpste24* (Bergo et al. 2002) were maintained on a pure C57BL/6N background and monitored daily and weighed weekly. As described previously, these mice appear normal during the first weeks of age, with a 5-10% lower body weight compared to wild-type animals. At 5-15 weeks old, mice show some signs of muscle weakness, and have a lower body weight than wild-type mice. Starting at 15 weeks of age, mice gradually lose weight, start to move more slowly, and appear more hunchbacked. Above 20 weeks, mice are less active, are wobbling or limping and keep losing weight. Mice have a lifespan of 20-35 weeks.

Mice, males and females, were randomly selected to be treated with either a vehicle or with Ruxolitinib. Ruxolitinib was administered through sub-cutaneous implantation of slow-release pellets (Innovative Research of America, Sarasota, FL, USA) of placebo or 30 mg/kg/day of Ruxolitinib, starting at 6 weeks of age. Two doses of pellets (18 mg or 30 mg of Ruxolitinib over 60 days) were designed to counterbalance the difference in weight between males and females. Mice were sacrificed when becoming moribund or when they had lost more than 20% of their weight over two consecutive weeks. Mouse experiments were approved by the Animal Research Ethics Committees in Gothenburg and Linköping, Sweden.

Phenotypical tests in mice

The ability of mice to hang for more than 30s on to an upside-down grid was assessed every week (yes or no assessment). Mice were considered as having an impaired grip when they were unable (no) to hang 2 weeks in a row. Bone mineral content and Bone mineral density were determined on whole body (with ROI excluding head and tail) and on hind legs stored in 70% ethanol by dual-energy X-ray absorptiometry (DEXA) using a Lunar PIXImus densitometer (Wipro, GE Healthcare).

Statistical analyses

Statistical analyses were performed using Microsoft Excel or GraphPad v 8.2. Statistical significance for mouse survival and grip strength was assessed by a Log-rank (Mantel Cox) test. All other parameters were analyzed with unpaired two-sided Student t-test. (* $P < 0.05$; ** $P < 0.01$; *** $P < 0.005$).
